# Food Choice Priorities Change Over Time and Predict Dietary Intake at the End of the First Year of College Among Students in the U.S.

**DOI:** 10.3390/nu10091296

**Published:** 2018-09-13

**Authors:** Melissa J. Vilaro, Sarah E. Colby, Kristin Riggsbee, Wenjun Zhou, Carol Byrd-Bredbenner, Melissa D. Olfert, Tracey E. Barnett, Tanya Horacek, Morgan Sowers, Anne E. Mathews

**Affiliations:** 1 Food Science and Human Nutrition Department, University of Florida, Gainesville, FL 32611, USA; mgraveley@ufl.edu; 2 Department of Nutrition, University of Tennessee, Knoxville, TN 37996, USA; Scolby1@utk.edu (S.E.C.); kristin.riggsbee@gmail.com (K.R.); 3 Department of Business Analytics and Statistics, University of Tennessee, Knoxville, TN 37996, USA; wzhou4@utk.edu (W.Z.); morganlfaulk@gmail.com (M.S.); 4 Department of Nutritional Sciences, Rutgers University, New Brunswick, NJ 08901, USA; bredbenner@sebs.rutgers.edu; 5 Animal and Nutritional Sciences Department, West Virginia University, Morgantown, WV 26506, USA; melissa.olfert@mail.wvu.edu; 6 School of Public Health, University of North Texas Health Science Center, Ft. Worth, TX 76107, USA; Tracey.Barnett@unthsc.edu; 7 Department of Public Health, Food Studies & Nutrition, Syracuse University, Syracuse, NY 13244, USA; thoracek@syr.edu

**Keywords:** food choice, dietary intake, college students, health behaviors, sugar-sweetened beverages, fruits and vegetables

## Abstract

This study assessed food choice priorities (FCP) and associations with consumption of fruits and vegetables (FV), fiber, added sugars from non-beverage sources, and sugar-sweetened beverages (SSB) among college students. Freshmen from eight U.S. universities (*N* = 1149) completed the Food Choice Priorities Survey, designed for college students to provide a way to determine the factors of greatest importance regarding food choices, and the NCI Dietary Screener Questionnaire. Changes in FCP and dietary intake from fall 2015 to spring 2016 were assessed. Multiple regression models examined associations between FCP and log-transformed dietary intake, controlling for sex, age, race, and BMI. Participant characteristics and FCP associations were also assessed. FCP importance changed across the freshmen year and significantly predicted dietary intake. The most important FCP were price, busy daily life and preferences, and healthy aesthetic. Students who endorsed healthy aesthetic factors (health, effect on physical appearance, freshness/quality/in season) as important for food choice, consumed more FV and fiber and less added sugar and SSB. Busy daily life and preferences (taste, convenience, routine, ability to feel full) predicted lower FV, higher added sugar, and higher SSB consumption. Price predicted lower FV, higher SSB, and more added sugar while the advertising environment was positively associated with SSB intake. FCP and demographic factors explained between 2%–17% of the variance in dietary intake across models. The strongest relationship was between healthy aesthetic factors and SSB (B = −0.37, *p* < 0.01). Self-rated importance of factors influencing food choice are related to dietary intake among students. Interventions that shift identified FCP may positively impact students’ diet quality especially considering that some FCP increase in importance across the first year of college.

## 1. Introduction

The traditional diet consumed by young adults in the United States is typically low in fruits, vegetables, legumes, and fiber, and high in added sugar, including sugars obtained from sugar-sweetened beverages (SSB) [1,2,3]. Unhealthy diets are risk-factors for multiple chronic diseases including obesity, type 2 diabetes, some cancers, and cardiovascular disease [4,5,6]. Among adults in 2012, low consumption of fruits and vegetables (FV) and excessive intake of SSB contributed to deaths due to heart disease (7.5%), stroke (7.6%), or type 2 diabetes (7.4%) [7]. 

The 2015–2020 Dietary Guidelines for Americans recommend eating a variety of vegetables, legumes, and whole fruits, choosing beverages without added sugars, and consuming less than 10% of calories per day from added sugars [8]. SSB include liquids sweetened with added sugars and can include but are not limited to sodas, fruit-flavored drinks, and sports drinks. Although, consumption of SSB is on the decline, adolescents are the most prevalent consumers compared to other age groups, with 65.4% of 12–19 year-olds drinking SSB in 2013–2014 [9]. Consumption of fruits, vegetables, and legumes, which are all important sources of dietary fiber, has been documented to decrease during the college years [10] and less than 5% of college students eat 5 or more servings of FV daily [11]. Thus, a deeper understanding of the drivers of food choice by college students will provide needed insights for effective nutrition promotion strategies. 

Conceptual models describing how adults choose foods detail the multidimensional nature of motivating factors that inform decisions regarding food for purchase or consumption. The Food Choice Process Model describes how values and behavioral strategies, informed by a person’s life-course experiences, guide food decisions [12] Food choice priorities typically described in the literature include factors such as price, health, taste, convenience, and social relationships [13,14]. Minority and lower-income populations more frequently cite non-health related drivers of food choice, indicating exploration of additional drivers of food choice may help mitigate diet-related disparities based on sociodemographic differences [13,15]. Aspects of the college lifestyle provide exposures that uniquely influence food choice decisions, including that many students are living independently for the first time and may access to dining halls and meal plans [16]. Specifically, college freshmen experiencing the transition to college are at risk of unwanted weight-gain during the first year [17,18]. Behaviors established during this life stage may persist into adulthood, making college an optimal period to launch life-long health behaviors [19]. 

### Study Objectives

The primary aim of this study was to examine the relationship between food choice priorities (FCP) and dietary intake among university students at the end of the freshman year. Secondary aims included describing how FCP vary based on demographic variables including sex, race, and body mass index (BMI), and to explore how FCP and dietary intake change over the course of the freshmen year of college.

## 2. Materials and Methods

### 2.1. Participants

Data were acquired as a component of a larger, prospective health-promotion study, Get FRUVED. As part of the project development phase, Get FRUVED enrolled first-year college students during summer and fall 2015, from eight universities in the United States. Each university’s respective Institutional Review Board approved the study protocol. Participants were eligible based on eating <2 cup equivalents (CE) of fruit and/or <3 CE of vegetables as measured by the National Cancer Institute’s (NCI) 9-item all-day screener [20], and having one additional self-reported, risk-factor for weight gain during the first year of college, including first-generation college student, BMI > 25 kg/m^2^, overweight or obese parent, racial/ethnic minority, or low-income status [21]. Eligibility criteria were selected based on the purpose of the larger study which was to improve FV intake and promote an overall healthy lifestyle among freshmen. Of the 5426 first-year students screened for eligibility, 2757 were eligible and 1149 were assessed at baseline. Participants completed web-based surveys and anthropometric measurements (height and weight) at fall 2015 (baseline) and spring 2016 (follow-up). The first year of the study was a development year with activities developed and implemented at four universities. Enrolled participants were invited but not required to participate in ongoing activities. Assessment procedures were identical at baseline and follow-up. 

### 2.2. Measures

#### 2.2.1. Food Choice Priorities (FCP) 

The Food Choice Priorities Survey (FCPS) asks participants to rate the importance of “the main factors that influence the food you eat on a regular basis” using a five-point Likert scale (1 = not important, 5 = extremely important). The FCPS was informed by interviews and focus groups with college students and assessed for content validity and reliability, which is described in full by Vilaro and colleagues [16] The FCPS contains three scales and five individual items. Scales include: food choice driven by the advertising environment containing two items, (social media (Pinterest, Instagram, other) and advertising (TV, magazines, other); food choice driven by a healthy aesthetic includes three items, (health, effect on physical appearance, and freshness/quality/in season); and food choice driven by busy daily life and preferences includes four items, (taste, convenience, routine/what I’m used to eating, and ability to feel full). The five individual FCPS items are: price, stress, family, peer and social situations, and boyfriend/girlfriend/significant other. FCP were utilized as independent variables in regression models.

#### 2.2.2. Dietary Intake 

The NCI Dietary Screener Questionnaire (DSQ) asks about frequency of intake in the past month for selected foods/drinks and provides daily intake values. Participants are asked to consider meals and snacks consumed at home, work, school, restaurants, and any other locations. Responses are converted to estimated intake values via scoring algorithms provided by the NCI [22]. We utilized intake values for fruit/vegetable/legumes minus French fries (FV) (cup equivalents (CE)/day), added sugar from sugar sweetened beverages (SSB) (tsp/day), added sugar from non-beverage sources (tsp/day), and fiber (g/day). Dietary intake was the dependent variable in regression models. 

#### 2.2.3. Anthropometrics

Weight was collected using digital scales and recorded to the nearest 0.1 kg. Height was collected using portable stadiometers and recorded to the nearest 0.1 cm. Measures were collected twice by trained researchers with a third measure required when differences between the first two measures exceeded 0.2 kg for weight or 0.2 cm for height. BMI was calculated from measured height and weight.

#### 2.2.4. Sociodemographic Variables 

Race, age [23], sex [24], and BMI [25] have been associated with dietary intake. Thus, in regression models, we controlled for these variable as potential confounders of the FCP-dietary intake relationship. 

### 2.3. Analysis 

Multiple linear regression models with simultaneous entry were used to determine the cross-sectional relationships between FCP and dietary intake at the end of the freshmen year. The end of the year was assessed to capture the cumulative effects of the first year of college. For each dietary intake variable, Model 1 included only FCP independent variables while Model 2 adjusted for age, race, sex, and BMI. Each dietary intake variable was log-transformed, using the natural log, to address violations of normality and equality of variances for residuals. To identify outliers, we analyzed saved Cook’s distances of the log-transformed variable. High-leverage, extreme observations that would influence overall models and predicted values were identified and removed based on typical cut-off points defined as a Cook’s distance greater than 4/n [26]. For FV, 47 outliers were excluded. Similarly, 36 outliers were excluded from added sugar, 29 outliers excluded from SSB, and 44 outliers excluded from fiber. 

For secondary aims, we analyzed changes in FCPS scores and dietary intake from baseline to follow-up using paired-sample *t*-tests. At baseline and follow-up, we assessed FCP differences by sex using independent sample *t*-tests and race with ANOVA and post-hoc tests. We also assessed associations between BMI and FCP with Pearson’s correlations. Statistical significance was set at *p* < 0.05. Analysis was performed using IBM SPSS for Windows (version 25.0. IBM Corp., Armonk, NY, USA). 

## 3. Results

Out of 1149 participants assessed at baseline, 857 (75%) completed follow-up assessments (Table 1). Based on Wilcoxon ranked sum tests, participants who attended follow-up assessments had lower BMI at baseline (M = 24.19, SD = 4.68) compared to those who did not attend follow-up (M = 25.08, SD = 5.28, *p* = 0.011). Chi square tests indicate females (78%) were more likely than males (69%) to complete follow-up assessments (*p* < 0.01). There was better retention of Hispanic (81.9%), black (78.6%), and multiracial students (74.2%) at follow-up compared to white students (72%, *p* < 0.05). 

At the follow-up assessment, 97% of students did not meet general recommendations for FV (5 CE/day) or fiber (25 g/day). Students consumed on average 220 calories/day of added sugar from non-beverage sources and 97 calories/day of added sugars from SSB based on an estimated 16 calories/tsp of sugar. Thus, based on an estimated 2500 calories/day, we identified students consuming >16.7g/day as above recommendations for added sugar (<10% of calories from added sugar/day) [8]. About 7% of students were above the recommended intake values for added sugars from SSB and 24% were above recommendations for added sugars from non-beverage sources. Additionally, at follow-up, almost 80% of students (680/855) indicated they were on a university meal plan for the previous 12 months.

### 3.1. Primary Aim: FCP and Dietary Intake at the End of the Freshmen Year

**Figure 1:**
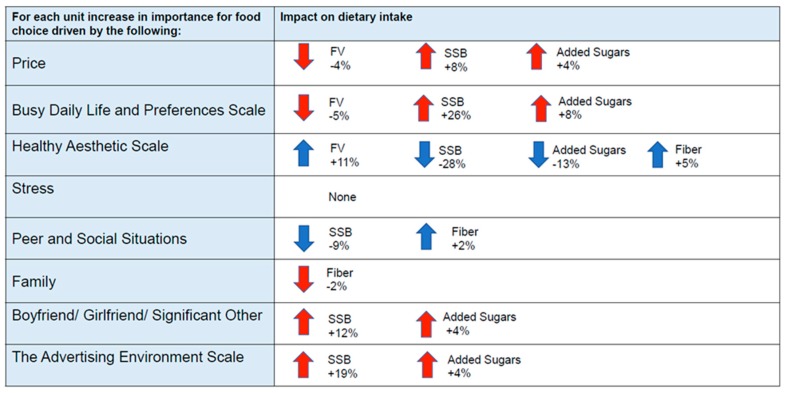
Cross-sectional, significant relationships between dietary intake and food choice priorities at the end of the freshmen year controlling for BMI, race, age, and sex (model 2). Numbers represent percentage change. Blue arrows indicate dietary intake in the direction one would prefer to promote healthy diets. Red arrows indicate dietary intake in a less desirable direction. Food Choice Priorities Survey (FCPS) scales and items are listed in order from most to least important based on mean ratings on a Likert scale. FV = Fruit and Vegetables minus French fries, SSB = Sugar sweetened beverages.

Sequentially adjusted linear regression models show relationships between FCP and consumption of FV, SSB, added sugars from non-beverage sources, and fiber (Table 2, Figure 1). Food Choice Driven by the Advertising Environment was significantly associated with increased consumption of SSB and added sugars such that as the advertising environment became more important for food choice, students consumed more SSB and added sugars. Food Choice Driven by a Healthy Aesthetic was positively associated with FV and fiber intake and negatively associated with consumption of SSB and added sugars. When Busy Daily Life and Preferences were more important for food choice, students consumed fewer FV, more SSB, and more added sugar, although added sugar was only significantly related to the advertising environment in Model 2 after controlling for demographic factors.

Individual FCPS items were also significantly associated with diet. As price became more important for food choice students consumed significantly fewer FV, more SSB, and more added sugars. The relationship between price and intake of SSB was significant only in model 2. As peer and social situations increased in importance students consumed less SSB and more fiber. The importance of a boyfriend/girlfriend/significant other when making food choices was associated with increased intake of both SSB and added sugars. When family was important for food choice students ate less fiber and this relationship was only found in Model 2. Stress was the only FCP not significantly related to dietary intake.

### 3.2. Sociodemographic Variables and Dietary Intake

Sociodemographic variables were control variables in Model 2. Male status was associated with statistically significant greater intake of all four measured components of dietary intake (FV, SSB, added sugar from non-beverage sources, and fiber) compared to females. As total energy intake was not controlled, this relationship was expected. BMI was inversely related to consumption of FV. Students who identified as multiracial consumed significantly less fiber and less added sugar compared to white students (Table 2).

### 3.3. Secondary Aim 1: Changes in FCPS Scores and Dietary Intake 

At baseline the three most important FCP were price (M = 3.63, SD = 1.11), busy daily life and preferences scale (M = 3.64, SD = 0.64), and the healthy aesthetic scale (M = 3.33, SD = 0.83), these remained the most important at follow-up. Price, busy daily life and preferences, and stress became significantly more important by the end of the freshmen year compared to baseline while the importance of family became significantly less important for food choice. The healthy aesthetic scale, advertising environment scale, peer and social situations, and boyfriend/girlfriend/significant did not significantly change in importance from baseline to follow-up. Mean intake of all four dietary variables (FV, SSB, added sugar from non-beverage sources, and fiber) significantly decreased from baseline to follow-up (Table 3). 

### 3.4. Secondary Aim 2: FCP and Participant Characteristics at Baseline and at Follow-up

#### 3.4.1. Sex

There were statistically significant differences regarding how males and females rated the importance of FCPS variables. At baseline and follow-up, females rated stress (M = 2.83, SD = 1.2; *t* (1120) = −6.81; M = 2.98, SD = 1.25; *t* (840) = −6.04) and the advertising environment (M = 1.73, SD = 0.94; *t* (941.1) = −5.09; M = 1.78, SD = 0.95; *t* (597.16) = −4.16) as significantly more important for food choices compared to males ratings of stress (M = 2.3, SD = 1.27; *t* (1120) = −6.81; M = 2.42, SD = 1.25; *t* (840) = −6.04) and the advertising environment (M = 1.47,SD = 0.73; *t* (941.1) = −5.09; M = 1.52, SD = 0.79; *t* (597.16) = −4.16). Healthy aesthetic factors were also more important for females at baseline and follow-up (M = 3.41, SD = 0.82; *t* (1120) = −4.27; M = 3.42, SD = 0.83; *t* (847) = −4.88) compared to males ratings of healthy aesthetic factors at both time points (M = 3.18, SD = 0.85; *t* (1120) = −4.27; M = 3.11, SD = 0.88; *t* (847) = −4.88), *p* < 0.001.

At the end of the freshmen year females rated price (M = 3.97, SD = 1.07; *t* (844) = −2.10) and the busy daily life and preferences scale (M = 3.73, SD = 0.67; *t* (847) = −2.09) more important compared to males rating of price (M = 3.80, SD = 1.14) and busy daily life and preferences scale (M = 3.63, SD = 0.67), *p* < 0.05. 

#### 3.4.2. Race 

At the start of the freshmen year, non-Hispanic black students’ food choices were more likely to be driven by busy daily life and preferences (M = 3.92, SD = 0.74) compared to non-Hispanic white (M = 3.61, SD = 0.63), multiracial (M = 3.64, SD = 0.67) and Hispanic/Latino students (M = 3.60, SD = 0.61), (F (3,1110) = 7.90, *p* = 0.014). Price was also significantly more important for black students’ food choices (M = 3.94, SD = 1.15) compared to both white students (M = 3.62, SD = 1.12) and Hispanic/Latino students (M = 3.58, SD = 1.09), (F (3,1110) = 3.08, *p* = 0.027). Peers and social situations were more important for white students’ food choices (M = 2.62, SD = 1.22) compared to black students (M = 2.23, SD = 1.24, (F (3,1110) = 3.55, *p* = 0.014).

At the end of the freshmen year, busy daily life and preferences was still more important for black students’ food choices (M = 3.93, SD = 0.83) compared to white students (M = 3.64, SD = 0.64) and Hispanic/Latino (M = 3.71, SD = 0.62), F (3839) = 5.32, *p* = 0.001]. The influence of a significant other was more important for white (M = 1.91, SD = 1.25) and multiracial students’ food choices (M = 1.97, SD = 1.16) compared to black students (M = 1.48, SD = 0.87), (F (3825) = 4.67, *p* = 0.003).

#### 3.4.3. BMI

At baseline, BMI (M = 24.39 kg/m^2^, SD = 4.85) was positively correlated with the following FCP; stress, *r* (1130) = 0.062, *p* = 0.038, family, *r* (1130) = 0.077, *p* = 0.009, the advertising environment, *r* (1130) = 0.073, *p* = 0.014, price *r* (1130) = 0.066, *p* = 0.026, and peer and social situations *r* (1130) = 0.139, *p* = 0.000. By the end of the freshmen year, at follow-up, BMI (M = 24.6 kg/m^2^, SD = 4.7) was positively correlated with stress, *r* (826) = 0.134, *p* = 0.000, family, *r* (830) = 0.074, *p* = 0.033 and peer and social situations, *r* (830) = 0.110, *p* = 0.002. 

## 4. Discussion

Food choice priorities (FCP), factors important for making decisions about foods consumed on a regular basis, are related to dietary intake of college freshmen. Students rated price, having a busy lifestyle, and health-related factors as the three most important FCP among students at both time points assessed. Three FCP (price, busy lifestyle and preferences scale, and stress) became more important drivers of food choice by the end of the freshmen year and only one FCP (family) became less important for food choice. While the healthy aesthetic scale did not significantly change in importance across the first year of college, it was associated with all four dietary components (FV, SSB, added sugar from non-beverage sources, and fiber) in a direction one would want to see for promoting healthy dietary intake. Stress was the only FCP not associated with dietary intake even though students rated it as more important for food choice at the end of the freshmen year compared to the start of the year. Additionally, various FCP were associated with race, sex, and BMI.

### 4.1. FCPS as Predictor of Healthy Dietary Intake

Not surprisingly, when food choice was driven by the healthy aesthetic scale, intake of healthier food components increased, and intake of unhealthy food components decreased, compared to students rating health as less important for food choice. Even though the healthy aesthetic scale was the third most important FCP, its mean level of importance remained the same at baseline and follow-up, indicating these factors may represent a stable FCP that are not readily changed. Females also rated healthy aesthetic factors as more important compared to males at both time points. Exploring the reasons behind endorsement of the healthy aesthetic FCP could inform its utility in the context of behavior change and dietary intake. For example, students may be internally motivated by a healthy aesthetic FCP desiring to present a healthy image to others. Alternatively, peers may be encouraging and modeling healthy behaviors providing an externally driven social norm contributing to this FCP. The external factors driving this FCP could also be less healthy norms such as weight preoccupation. 

The influence of the FCP peer and social situations appears beneficially impact diet quality of university students. As peers and social situations was rated more important for food choice, students consumed fewer SSB and more fiber. Although findings indicated a protective effect of peers and social situations for dietary intake, the nuances between when social interactions may be helpful for promoting healthy diet or act as a barrier to healthy intake is still not fully understood. Consistent with our study, other reports have demonstrated social relationships may facilitate healthy eating [27]. However, social eating has also been associated with less healthy dietary intake [28] possibly due to environmental cues that promote unhealthy eating habits [29]. In one study, greater intake of SSB by peers was associated with more SSB consumption among friends unless peers thought healthy eating was important; then SSB intake among friends declined [30]. Our data seems to suggest first-year students may be experiencing positive cues from dynamics that emerge when eating socially indicating students’ peer-networks and social eating occasions may present opportunities to promote healthful dietary intake. Additional studies with study designs addressing these nuanced aspects are needed to clarify relationships.

### 4.2. FCPS as Predictor of Less Healthy Dietary Intake

In contrast to the seemingly healthy impact of social situations and peers on dietary intake, rating a significant other as an important influence on food choice may play a less supportive role. As a significant other became more important regarding food choices, students consumed more SSB and more added sugar from non-beverage sources, highlighting the different effect of two types of social relationships for diet (peers vs. a significant other). Family, another source of social input, was related to consuming less fiber. Family was also the only FCP to significantly decrease in importance by the end year, a decline that makes sense given that students are likely no longer living with or spending as much time with family as they were prior to attending college. These findings indicate future research is needed to distinguish between different types of social relationships and explore the mechanisms by which relationships are supportive or have consequences for dietary intake.

When food choices were driven by busy daily life and preferences scale students ate fewer FV, more SSB, and more added sugar indicating that the college lifestyle and personal preferences may be negatively impacting students’ abilities to consume healthy diets. Some of the items comprising the busy daily life and preferences scale, in particular taste and routine/what I’m used to eating, may be tapping into a related construct known as picky eating. Picky eating has been assessed in other studies with the Adult Picky Eating Questionnaire (APEQ) [31]. Similar to our findings with busy daily life and preferences FCP, picky eating has also been associated with lower amounts and variety of FV intake among college students [31,32]. Students valuing convenience may be making time-saving choices that translate to pre-packaged foods that are higher in added sugar. In one study, students who were influenced by hunger, taste, friends, media, time and budget consumed more energy and more fat compared to students whose dietary behaviors were more influenced by health and weight [33]. Thus, addressing components of the college lifestyle, convenience, and individual eating preferences could help prevent a decline in diet quality among students.

Busy daily life and preferences scale was significantly more important for black students compared to students from other racial backgrounds at both baseline and follow-up and was positively related to higher SSB and added sugar intake as well as eating fewer FV. Our results are notable given recent findings indicating between 2003 and 2014, there was an overall decrease in SSB consumption trends, with significant declines in all age categories of non-Hispanic white participants across 6 distinct age categories (ages 2–60+ years) however there were no significant changes among 2 age categories of non-Hispanic black participants (6–11 or 20–39 years) [9]. Bleich and colleagues [9] also found non-Hispanic black, Mexican American, and non-Mexican Hispanics represent the largest percentage of SSB consumers, with non-Hispanic black children aged 12–19 years old representing the largest group (78.3% consuming SSB). These findings suggest busy daily life and preferences priorities may be of importance among students who identify as black, specifically regarding SSB. Engaging diverse youth in efforts to understand and navigate barriers related to busy college lifestyle and preferences are warranted to help promote healthy dietary intake.

Additionally, our findings indicate as price became more important for food choice students consumed fewer FV, more SSB, and more added sugars. Price was the most important FCP and significantly increased in importance by the end of the freshmen year, indicating price may warrant special attention as an important driver of unhealthy food choices. Other studies also consistently cite price as an important consideration for food choices [34,35] This FCP was also significantly more important for black students compared to others, but only at the start of the year, indicating a point of intervention that should be explored to address diet-related health disparities that may in part be driven by financial concerns.

The advertising environment was rated as the least important FCP, however as it became more important, students consumed more SSB and more added sugar. People tend to underestimate the effect advertising on themselves. The Perceptual Hypothesis in advertising explains why people perceive themselves as less susceptible to advertising compared to others, although this can vary based on type and content of messages [36]. Our findings are also consistent with studies showing unhealthy food advertising exposure has been associated with increased consumption of junk food [37,38,39] and frequent consumption of SSB is associated with more screen time among adolescents [40]. Even though the advertising environment was perceived as not important for food choice the pervasiveness of food advertising among youth in the U.S. [41] and our findings indicate advertisements play a role in dietary intake, specifically sugar, among students. 

### 4.3. Limitations

Dietary intake and FCP were assessed using validated tools, however self-reported dietary intake can be subject to error and demonstrate limitations among persons of different weight statuses, sex, and literacy levels [42,43,44,45]. Additionally, participants were eligible to enroll based on low FV intake plus one additional risk-factor for unwanted weight gain, making results not generalizable to all college students. Last, self-rated importance may not capture all factors that influence food choice, given individuals may be unaware of all driving factors, as may be the case with the role of advertising.

## 5. Conclusions

FCPS scores predict dietary patterns of college freshmen. Busy lifestyles and financial concerns are common components of the college experience and among the most important FCP identified. Dietary and behavioral interventions should address these factors to promote healthy diets among students. The positive impact of peers and social situations on dietary intake signals a potential protective effect of the peer dynamic that warrants further research. The importance of food choice driven by a healthy aesthetic did not change in importance by the end of the first year, implying that interventions solely focused on health may only be appropriate for students who are already receptive to health messages. 

## Figures and Tables

**Table 1:** Participant Characteristics.

(M, SD)	Fall 2015	Spring 2016
	*N* = 1149	*n* = 857
BMI (kg/m^2^)	24.4 (4.9)	24.6 (4.7)
(n, %)		
Female	745 (64.8)	585 (68.5)
Male	377 (32.8)	265 (31.0)
18 years old	974 (84.6)	293 (34.7)
19 years old	128 (11.1)	520 (61.5)
20 years old	11 (1.0)	18 (2.1)
21+ years old	14 (1.3)	14 (1.7)
Non-Hispanic White	604 (52.5)	433 (51.3)
Non-Hispanic Black	117 (10.2)	86 (10.2)
Hispanic/Latino only	204 (17.8)	170 (20.1)
Other or multiracial	190 (16.5)	155 (18.4)
Live on campus	957 (83.3)	704 (82.5)
Live off campus	133 (11.5)	117 (13.7)
Live with parents	21 (1.8)	16 (1.9)
Live in sorority/fraternity	11 (1.0)	12 (1.4)
Other living arrangement	4 (.3)	1 (.1)
Alabama	81 (7.0)	57 (6.7)
Florida	299 (26.0)	244 (28.5)
Maine	167 (14.5)	130 (15.2)
Kansas	111 (9.7)	94 (11.0)
New York	156 (13.6)	130 (15.2)
Tennessee	171 (14.9)	88 (10.3)
South Dakota	69 (6.0)	44 (5.1)
West Virginia	95 (8.3)	70 (8.2)
Currently single	752 (65.4)	538 (63.1)
Currently in a relationship	348 (30.3)	301 (35.3)

M = mean, SD = standard deviation, BMI = body mass index.

**Table 2:** Food Choice Priorities Scale predictors of dietary intake at the end of the first year of college.

	Model 1			Adjusted Model 2 (Age, Race, Sex, BMI)		
	B	SE	*p*	B	SE	*p*
Fruits, vegetables, legumes (CE/day) *				−0.01	0.02	0.51
Advertising Environment Scale	−0.02	0.16	0.20	0.10	0.16	0.00
Healthy Aesthetic Scale	0.09	0.16	0.00	−0.05	0.02	0.04
Busy Daily Life and Preferences Scale	−0.05	0.02	0.02	−0.04	0.01	0.00
Price	−0.04	0.01	0.00	0.01	0.01	0.43
Stress	−0.01	0.01	0.52	−0.02	0.01	0.11
Family	−0.02	0.01	0.10	0.01	0.01	0.52
Peer	0.02	0.01	0.23	0.00	0.01	0.87
Significant other	0.00	0.01	0.83	0.16	0.03	0.00
Sex (ref = Female)	NA			0.00	0.02	0.92
Age	NA					
Race (ref = White)				−0.03	0.05	0.49
Black	NA			−0.03	0.03	0.35
Hispanic	NA			−0.03	0.04	0.34
Multiracial	NA			−0.01	0.00	0.04
BMI	NA					0.10
Adj. R^2^			0.06			
**Added sugar from sugar sweetened beverages (tsp/day)**				0.18	0.05	0.00
Advertising Environment Scale	0.13	0.05	0.01	−0.33	0.05	0.00
Healthy Aesthetic Scale	−0.37	0.05	0.00	0.23	0.07	0.00
Busy Daily Life and Preferences Scale	0.23	0.07	0.00	0.08	0.04	0.04
Price	0.06	0.04	0.10	−0.01	0.04	0.72
Stress	−0.04	0.04	0.25	0	0.04	0.90
Family	0.02	0.04	0.63	−0.09	0.04	0.03
Peer	−0.09	0.04	0.02	0.11	0.04	0.00
Significant other	0.13	0.04	0.00			
**Added sugar from sugar sweetened beverages (tsp/day)**				0.57		
Sex (ref = Female)	NA			0.05	0.09	0.00
Age	NA				0.05	0.33
Race (ref = White)				0.09		
Black	NA			−0.11	0.14	0.51
Hispanic	NA			−0.18	0.10	0.28
Multiracial	NA			0.01	0.12	0.10
BMI	NA				0.01	0.43
Adj. R^2^			0.09			0.14
**Added sugar (tsp/day)**				0.04		
Advertising Environment Scale	0.02	0.02	0.22	−0.14	0.02	0.03
Healthy Aesthetic Scale	−0.16	0.02	0.00	0.08	0.02	0.00
Busy Daily Life and Preferences Scale	0.07	0.03	0.00	0.04	0.03	0.00
Price	0.03	0.01	0.045	0.01	0.01	0.38
Stress	0.00	0.01	0.87	0.01	0.01	0.30
Family	0.02	0.01	0.2	0.01	0.01	0.19
Peer	−0.02	0.02	0.28	−0.02	0.02	0.01
Significant other	0.04	0.01	0.00	0.04	0.01	0.00
Sex (ref = Female)	NA			0.26	0.03	0.37
Age	NA			0.02	0.02	
Race (ref = White)						0.21
Black	NA			−0.07	0.05	0.27
Hispanic	NA			−0.04	0.04	0.00
Multiracial	NA			−0.13	0.04	0.76
BMI	NA			0.00	0.00	0.17
Adj. R^2^			0.10			
**Fiber (g/day)**						
Advertising Environment Scale	−0.02	0.01	0.2	−0.01	0.01	0.62
Healthy Aesthetic Scale	0.03	0.01	0.01	0.05	0.01	0.00
Busy Daily Life and Preferences Scale	−0.03	0.02	0.08	−0.02	0.02	0.23
Price	−0.01	0.01	0.1	−0.01	0.01	0.24
Stress	−0.01	0.01	0.27	0.01	0.01	0.30
Family	−0.01	0.01	0.08	−0.02	0.01	0.04
Peer	0.02	0.01	0.05	0.02	0.01	0.05
Significant other	0.00	0.01	0.65	0.00	0.01	0.70
Sex (ref = Female)	NA			0.22	0.02	0.00
Age	NA			0	0.01	0.75
Race (ref = White)						
Black	NA			−0.06	0.03	0.07
Hispanic	NA			−0.03	0.02	0.14
Multiracial	NA			−0.04	0.02	0.00
BMI	NA			0	0.00	0.08
Adj. R^2^			0.02			0.16

Estimates obtained from multiple linear regression models run separately for each outcome. NA = not included in model. * Fruits, vegetables, legumes variable does not include French fries and is measured in cup equivalents/day (CE/day). Parameter estimates for all dietary outcome variables were calculated using the natural log transformed values with outliers removed. Model 1 = unadjusted with FCPS items as IV only, Model 2 = adjusted for sex, age, race, and BMI.

**Table 3:** Dietary intake and food choice priorities ratings at the beginning and end of the freshmen year of college.

Mean (*SD*)	2015	2016	*t* Statistic (*df*)
Dietary Intake			
Fruit/vegetable/legumes minus French fries (CE/day)	2.49 (*1.12*)	2.26 (*0.98*)	5.91 (832) ***
Added sugar from sugar sweetened beverages (tsp/day)	7.32 (*8.85*)	6.07 (*8.04*)	4.32 (833) ***
Added sugar (tsp/day)	15.11 (*8.39*)	13.72 (*7.78*)	5.30 (780) ***
Fiber (g/day)	14.73 (*5.84*)	13.65 (*4.54*)	6.19 (779) ***
Food Choice Priorities Survey Scales/Items			
Price	3.63 (*1.11*)	3.92 (*1.09*)	−7.70 (846) ***
Busy Daily Life and Preferences (Scale)	3.64 (*0.64*)	3.71 (*0.67*)	−2.90 (849) **
Healthy Aesthetic (Scale)	3.33 (*0.83*)	3.33 (*0.86*)	.31 (849)
Stress	2.62 (*1.23*)	2.80 (*1.28*)	−4.00 (842) ***
Peer and social situations	2.53 (*1.20*)	2.55 (*1.22*)	−0.58 (846)
Family	2.62 (*1.26*)	2.43 (*1.28*)	3.91 (846) ***
Boyfriend/girlfriend/significant other	1.80 (*1.13*)	1.85 (*1.18*)	−0.98 (835)
Advertising Environment (Scale)	1.65 (*0.86*)	1.70 (*0.91*)	−1.80 (845)

Paired *t*-tests were conducted to assess changes between start of freshmen year (2015) and end of freshmen year (2016). *** *p* < 0.001, ** *p* < 0.01. Food Choice Priorities assess importance of items for food consumed on a regular basis (1 = not important; 5 = extremely important).
